# Coupling droplets/bubbles with a liquid film for enhancing phase-change heat transfer

**DOI:** 10.1016/j.isci.2021.102531

**Published:** 2021-05-11

**Authors:** Rongfu Wen, Wei Liu, Xuehu Ma, Ronggui Yang

**Affiliations:** 1 State Key Laboratory of Fine Chemicals, Liaoning Key Laboratory of Clean Utilization of Chemical Resources, School of Chemical Engineering, Dalian University of Technology, Dalian 116024, China; 2 School of Energy and Power Engineering, Huazhong University of Science and Technology, Wuhan 430074, China

**Keywords:** Heat transfer, Phase-change, Interface transport, Fluid dynamics, Energy systems

## Abstract

Evaporation, boiling, and condensation are fundamental liquid-vapor phase-change heat transfer processes and have been utilized in many conventional and emerging energy systems. Recent advances in the manipulation of interface wetting and heterogeneous nucleation using micro/nano-structured surfaces have enabled exciting two-phase flow dynamics and heat transfer enhancement. However, independently manipulating droplets, bubbles, or liquid films through surface modification has encountered bottlenecks. In this Perspective, we discuss an emerging strategy where droplets/bubbles are coupled with a liquid film to control fluid dynamics for minimizing the thermal resistance between the liquid-vapor interface and solid substrate, thus significantly enhancing the heat transfer performance beyond the state of the art.

## Highlights

• Phase-change heat transfer enhancement is essential in many emerging energy systems.
• Independently manipulating droplet/bubble/liquid film has encountered bottlenecks.
• An emerging strategy of coupling droplet/bubble with a liquid film is discussed.
• Opportunities and challenges remain for further exploring the coupling strategy.

## Graphical abstract

**Figure:**
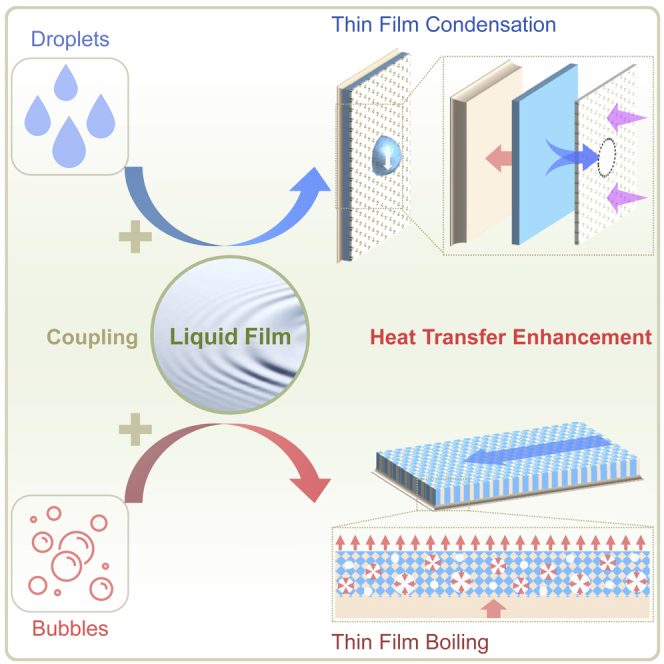

## Introduction

**Figure 1:**
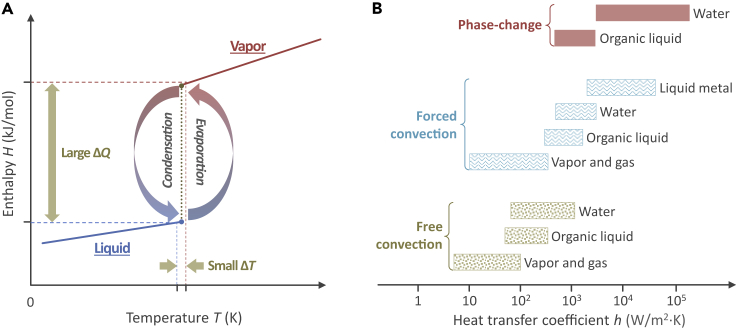
Liquid-vapor phase-change heat transfer and heat transfer coefficients (HTCs) of phase-change and single-phase convection heat transfer (A) Liquid-vapor phase-change processes: A large amount of latent heat is absorbed or released under a small temperature difference during the phase transition of the fluids. (B) Comparison of the HTCs for single-phase convection and phase-change heat transfer processes. Due to a large amount of absorbed or released latent heat, the HTCs of liquid-vapor phase-change processes far exceed that of single-phase convection heat transfer.

Heat transfer has been at the forefront of both fundamental and engineering research for more than a century due to its significance in many industrial applications, such as power generation, metallurgy and metal forming, heating and air conditioning, food processing, water treatment and purification, and thermal management of electronics (Cho et al., 2016; Chu and Majumdar, 2012; Elimelech and Phillip, 2011; Fan, 2017; Jiang and Singamaneni, 2017; Schelling et al., 2005; Wen et al., 2015). The fundamental heat transfer modes can be characterized as “conduction” where thermal energy is transferred from a hot end to a cold end due to the ubiquitous molecular/lattice vibration and/or electron motion, and “thermal radiation” where thermal energy is transmitted in the form of electromagnetic waves (Bergman et al., 2011). Between a solid surface and an adjacent fluid that is in motion, the thermal energy is transferred in “convection” mode, which results from the combined effect of thermal conduction and fluid motion. Compared with these single-phase heat transfer modes that primarily manifest themselves as large temperature differences, liquid-vapor phase-change heat transfer processes, *e.g.*, evaporation, boiling, and condensation, are much more efficient for energy transport due to the absorption or release of a large amount of latent heat under a small temperature difference during phase transition of the fluid (Figure 1A). Evaporation/boiling and condensation are critical for the heat engines in power generation and utilization systems that rely on liquid-vapor phase-change cycles, *e.g.,* thermoelectric power plants and air conditioners, and for dissipating high-flux waste heat from a small area, *e.g.*, electronics cooling applications (Cho et al., 2016; Wen et al., 2018a). The equivalent heat transfer coefficients (HTCs) of phase-change processes for the water, *e.g.*, condensation and boiling, can reach a value larger than 10^5^ W/m^2^K, which far exceeds that of the single-phase natural and forced convection heat transfer (Figure 1B) (Mudawar, 2001). Indeed, the reliability of nuclear power plants highly depends on the performance of two-phase boiling heat transfer, and the size of the condensers for both power plants and air conditioners are determined by the efficiency of condensation heat transfer.

Heat transfer performance of liquid-vapor phase-change processes is strongly affected by the wetting behaviors and flow dynamics of a fluid on a solid surface, where a droplet, a bubble or a liquid film is formed during the phase transition of the fluid (Allara, 2005; Cho et al., 2016; Wen et al., 2016, 2018a, 2019). Recent advances in micro/nano-fabrication and functional coatings have not only enabled interesting interfacial phenomena of droplet/bubble dynamics and liquid film spreading but also led to exciting improvements in heat transfer performance (Allred et al., 2018; Boreyko and Chen, 2009; Cheng et al., 2019; Cho et al., 2016; Dai et al., 2018; Daniel et al., 2001; Dhillon et al., 2015; Park et al., 2016; Wen et al., 2017b). The critical heat flux (CHF) and HTC of phase-change heat transfer on the micro/nano-structured surfaces have been increased to several times higher than that of the smooth surface under some specific conditions, *e.g.*, enhanced boiling CHFs of 200-400 W/cm^2^ and condensation HTCs of 100 kW/m^2^K (Li et al., 2019; Wen et al., 2017a). Most of the recent strategies have been focused on reducing the effective thermal resistance of the fluids in contact with the solid surface, *e.g.*, minimizing droplet departure size by reducing surface adhesion in dropwise condensation (Enright et al., 2014; Park et al., 2016; Rose, 2002; Wen et al., 2018d), thinning liquid film by promoting liquid spreading in capillary evaporation (Ćoso et al., 2012; Plawsky et al., 2014), and increasing convective liquid flow by accelerating bubble departure from a heated surface in pool boiling (Angulo et al., 2020; Narezo Guzman et al., 2015; Yarin, 2006). Despite significant improvements in heat transfer performance reported over the last couple of decades, independent manipulation of droplets, bubbles, or liquid films also encounters some emerging challenges due to the inherent complexity of multiscale phase-change processes. In this Perspective, we discuss an emerging strategy to enhance phase-change heat transfer that can break the limit and significantly advance the field, by coupling droplets/bubbles with a liquid film to minimize the effective thermal resistance from the phase-change interface to the solid surface.

### Condensation heat transfer enhancement

**Figure 2:**
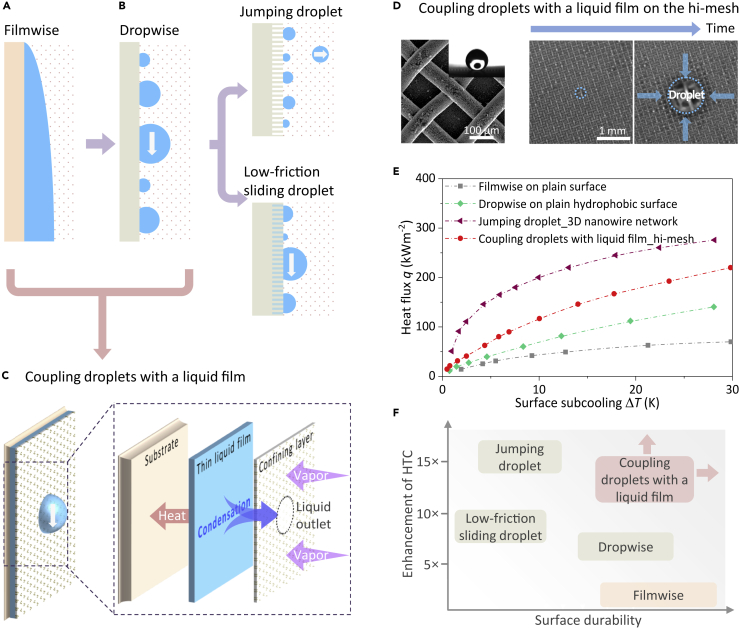
Condensation modes and heat transfer performance (A) Filmwise condensation on a vertical hydrophilic surface. (B) “Conventional” dropwise condensation on a vertical hydrophobic surface and its recent advances: self-propelled jumping droplets on a nanostructured surface and low-friction sliding droplets on an infused-lubricant structured surface. (C) Scheme to enhance condensation by coupling droplets with a liquid film: A thin condensate film is limited by a confining vapor-permeable layer. The vapor-permeable layer is non-wetting to liquid condensate but highly permeable to the vapor. Condensate film is thus confined between the substrate and confining layer. Liquid condensate is then removed in the form of falling droplets from the liquid outlets distributed on the confining layer. (D) Demonstrated droplet-liquid film coupling on a vertical hierarchical mesh-covered (hi-mesh) surface. Vapor permeates through the mesh layer and condenses on the substrate to form a thin liquid film that eventually leaves the surface in the form of gravity-driven falling droplets. Reprinted with permission from (Wen et al., 2018c). Copyright (2018) Oxford University Press. (E) Sustainably enhanced condensation for the coupling scheme was demonstrated in recent experiments where the condensate film thickness is confined by hi-mesh when compared with conventional filmwise and dropwise condensation (Wen et al., 2018c). Published by Oxford University Press. (F) Comparison of HTC and surface durability for different condensation enhancement schemes.

Depending on the wetting behavior and flow dynamics of a liquid condensate on a surface, condensation mode is characterized as filmwise condensation with the formation of a continuous liquid film on a high surface energy hydrophilic substrate (Figure 2A) and dropwise condensation with discrete droplets on a low surface energy hydrophobic surface (Figure 2B) (Ma et al., 2000; Rose, 2002). The condensate film that accumulates and grows in thickness along the gravitational direction, commonly acts as a large thermal barrier to hinder vapor condensation. Filmwise condensation can be enhanced by extending solid surface area or increasing interface fluctuation of condensate film, *e.g.*, fins and external fields, resulting in up to a 400% enhancement in HTC for water at atmospheric pressure (Rose, 2006, 2017; Wen et al., 2019). However, the most recent literature has focused on promoting dropwise condensation by surface modification, *e.g.*, low surface energy non-wetting coatings and low-friction lubricant-infused surfaces (Boreyko and Chen, 2009; Cho et al., 2016; Dai et al., 2018; Gong et al., 2017; Hou et al., 2015; Khalil et al., 2019; Lo et al., 2019; Ma et al., 2020; Miljkovic et al., 2013; Park et al., 2016; Preston et al., 2015; Rose, 2020; Sett et al., 2019; Wen et al., 2016, 2017b, 2017c, 2018a, 2018d). During “conventional” dropwise condensation, droplets fall off from the surface due to gravity when the droplet diameter grows up to a critical diameter comparable to the capillary length, *e.g.*, 2.7 mm for water (Wen et al., 2018a). Compared with filmwise condensation, dropwise condensation is more desirable since the HTC can be 5–7 times higher due to the much smaller effective thermal resistance between liquid-vapor interface and solid surface as the falling droplets frequently refresh the solid surface (Cho et al., 2016; Rose, 2020). To achieve dropwise condensation, non-wetting hydrophobic promoters or coatings have been widely used to alter the effective contact angle and surface adhesion of metallic surfaces, for more than nine decades since its discovery (Holden et al., 1987; Rose, 2002). About a decade ago, the low-friction droplet sliding (with a diameter of 1.5–2 mm) on the lubricant-infused surfaces and the self-propelled droplet jumping (with a droplet diameter smaller than 100 μm) on the micro/nano-structured non-wetting surfaces were discovered, which have revitalized the interests in promoting droplet dynamics for dropwise condensation enhancement (Figure 2B) (Boreyko and Chen, 2009; Wen et al., 2018a; Wong et al., 2011). In addition to hydrophobic coatings and micro/nanostructures, hydrophilic surfaces with low contact angle hysteresis present an opportunity to achieve dropwise condensation by promoting a high degree of chemical and physical homogeneity (Cha et al., 2020) or tethering flexible polymers with gradient grafting density (Zhang et al., 2021). Intensive efforts have been devoted to both the experimental demonstration of heat transfer enhancement due to accelerated droplet removal and the scaling-up of such an interesting interfacial phenomenon for useful thermal management devices (Cho et al., 2016; Ma et al., 2020; Miljkovic et al., 2013; Park et al., 2016; Wen et al., 2017c, 2018a, 2018d; Wiedenheft et al., 2017). However, further heat transfer enhancement in dropwise condensation has encountered bottlenecks by just minimizing droplet departure size or accelerating surface refreshing frequency. For example, an additional thermal barrier of low thermal conductivity air pockets or lubricant layer is inevitably introduced under condensed droplets on the non-wetting or lubricant-infused surfaces, which discounts the benefits of reduced thermal resistance of small droplets (Miljkovic et al., 2012; Wen et al., 2018d). In addition, the lack of thin hydrophobic coatings or stable lubricant layers with long-term durability and low thermal resistance makes the industrial implementation of dropwise condensation difficult (Holden et al., 1987; Ma et al., 2020; Rose, 2002).

The key to improving condensation heat transfer is to minimize the overall thermal resistance from the vapor condensation front, *e.g.*, the surface of condensed droplets, to the solid surface. As such, it is desirable to decrease the conduction thermal resistance of liquid condensate while not introducing an additional thermal barrier layer under the droplets (Jing and Guo, 2019; Miljkovic et al., 2012). Compared with the high-mobility droplets that have been pursued in the past several decades for enhancing dropwise condensation, a liquid film is more naturally condensed on a plain surface, *e.g.*, copper, aluminum, stainless steel, and silicon, without requiring additional low surface energy coatings and lubricant layers (Oh et al., 2018; Preston et al., 2018; Wen et al., 2018a). However, the performance of “conventional” filmwise condensation is limited, which is due to the thick and continuous liquid condensate film (up to 100 μm) with low thermal conductivity (0.6 W/mK for water). If such a liquid condensate film is thinned to dozens of micrometers or even only a few micrometers, the conduction thermal resistance of condensate film can be greatly reduced, especially when a liquid condensate-solid composite layer with a low thermal barrier is formed on a superhydrophilic porous surface. Benefiting from the high thermal conductivity of the porous surfaces such as metal foams and copper meshes (Preston et al., 2018; Wen et al., 2018a), the effective thermal conductivity of liquid condensate-solid composite layer can be increased up to several tens of times that of pure condensate film without additionally thickening the condensate film. The HTC of such thin film condensation on the porous surface can thus be increased by an order of magnitude, comparable to that observed during dropwise condensation (Oh et al., 2018; Preston et al., 2018; Wang and Antao, 2018). However, liquid condensate can flow over the top of the porous surface under large surface subcooling or condensation heat flux where more liquid condensate needs to flow through the porous structure, resulting in an added thermal resistance of flooded liquid film.

To minimize the film thickness and suppress the overflowing of liquid condensate simultaneously, a low surface tension membrane that is non-wetting to liquid condensate and highly permeable to vapor flow is proposed to confine condensate film formed on the substrate (Figure 2C). The Laplace pressure barrier (Kreit et al., 2010) that is formed by the wettability difference between non-wetting layer and high-wetting substrate can press liquid condensate inside the porous substrate to prevent overflowing of liquid condensate (Anderson et al., 2012; Oh et al., 2018), especially for high condensation heat flux and large condensation area. During condensation, the vapor can freely flow onto the cold substrate through high-permeability porous membrane and rapidly condense on the high surface tension substrate to release latent heat, forming a thin liquid film between the confining membrane and the substrate. The composite layer of the liquid condensate-porous structure increases the effective thermal conductivity compared with pure liquid film in conventional filmwise condensation, resulting in a smaller conduction thermal barrier for vapor condensation. Subsequently, the condensate is continuously removed in the form of thin liquid film flowing through the porous structure by gravity at low condensation heat flux (or surface subcooling) (Oh et al., 2018; Preston et al., 2018). With the increase of heat flux, more liquid condensate needs to be drained out from the confining membrane. Physically, as the surface subcooling increases up to a critical value, the Laplace pressure barrier is not enough to completely limit liquid condensate film underneath the confining membrane (Preston et al., 2018; Wang and Antao, 2018). To prevent the uncontrolled overflow of condensate film to maintain a thin film, liquid condensate outlets with larger pore sizes are distributed on the permeable membrane for liquid condensate drainage. Once condensate film pressure increases up to larger than the pressure barrier of the distributed liquid outlets, liquid condensate can rush out and thus be removed through the outlets as falling droplets (Figure 2C). Such a coupling mechanism of falling droplets with a composite layer of the liquid condensate-solid porous structure presented here is to enhance condensation heat transfer of a thin liquid film with the assistance of droplet draining. This is fundamentally different from the previous work using hybrid hydrophobic-hydrophilic surfaces with stripes and Mazak patterns to enhance dropwise condensation by sacrificing local filmwise condensation (Bintein et al., 2019; Lo et al., 2019; Peng et al., 2015; Winter and McCarthy, 2020).

This coupling scheme was recently reported on a hierarchical mesh-covered (hi-mesh) surface (Wen et al., 2018c). The typical structural feature of the hi-mesh surface is composed of an interweaving microchannel network between a superhydrophobic woven mesh layer and a flat substrate, as well as a large number of micropores among mesh wires (Figure 2D). Vapor permeates freely through the mesh layer and condenses on the cold substrate to form a thin liquid condensate film that is confined in the interconnected channel network. When the condensate film grows out of the micropores under large surface subcooling where the condensate film pressure is larger than the pressure barrier of the micropores, the surrounding liquid condensate is drained out by the liquid film sucking flow and eventually leaves the hi-mesh surface in the form of gravity-driven falling droplets. The thin liquid film in the interweaving microchannel network not only provides a low thermal resistance condensation interface but also continuously transports liquid condensate to be drained out from the substrate. Fourfold higher droplet growth rate and more than a third higher surface refreshing rate have been demonstrated experimentally, leading to a sustainable heat transfer enhancement on the hi-mesh surface when compared with conventional filmwise and dropwise condensation (Figure 2E) (Wen et al., 2018c). Note that condensation heat transfer data collected by different researchers are usually under various experimental conditions, *e.g.*, type of fluids, pressure and flow rate of the vapor, and NCG concentration (Miljkovic et al., 2013; Peng et al., 2015; Wen et al., 2017c). Figure 2E shows the comparison of condensation heat transfer data under the same operating conditions, 60 kPa water vapor with the concentration of NCGs smaller than 2.5%.

In the broader context of liquid condensate manipulation for enhancing condensation heat transfer, surface durability is a fundamental bottleneck that limits the implementation and deployment of dropwise condensation since its discovery in the early 1930s (Figure 2F). Compared with filmwise condensation on a plain surface, an additional hydrophobic coating that renders dropwise condensation, *e.g.*, conventional dropwise condensation and jumping droplet condensation, is a commonly non-polar organic material, which has a low intrinsic thermal conductivity of 0.1–1 W/mK. To preserve the heat transfer advantage of dropwise condensation, the hydrophobic coating needs to be ultra-thin, such as polymeric films thinner than 1 μm or self-assembled monolayers in about 1 nm thick (Ma et al., 2020). However, these thin hydrophobic coatings suffer from desorption-induced chemical instability or nucleation-induced coating delamination over time in the environments of dropwise condensation (Ma et al., 2019; Wen et al., 2018a). The additional micro/nanostructures or lubricant-infused layers further decrease surface durability and increase the difficulty to scale up cost-effectively for jumping droplet condensation and low-friction sliding droplet condensation (McCarthy et al., 2014; Preston and Wang, 2018). Compared with the requirements of ultra-thin non-wetting coatings or lubricant-infused surfaces for dropwise condensation, surface robustness is a significant advantage of thin film condensation on the porous structure. Note that the high-permeability membrane needs not be reduced to nanoscale thickness because it does not act as a direct conduction thermal barrier under liquid condensate. The commercial porous materials, *e.g.*, copper meshes and foams, and porous non-wetting layer, *e.g.*, PTFE membrane, provide a low-cost solution to manipulate liquid films and droplets on the constructed surfaces with enhanced condensation heat transfer (Wen et al., 2018c).

### Evaporation/boiling heat transfer enhancement

**Figure 3:**
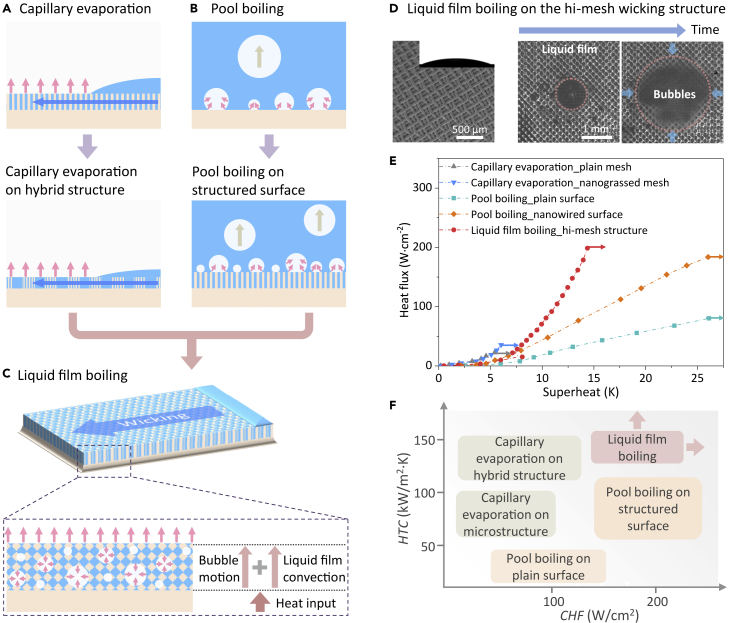
Evaporation/boiling and their heat transfer performance (A) Evaporation on capillary wicking structures. Hybrid wicking structures are used to enhance evaporation by reducing the thickness of capillary liquid film with reduced flow resistance. (B) Nucleate boiling heat transfer in a liquid pool. Micro/nano-structured surfaces can enhance nucleate boiling by both increasing nucleation sites and increasing the liquid wicking capability. (C) Strategy in enhancing evaporation through liquid film boiling: By introducing dynamic bubbles into a liquid film in the capillary wicking structure, heat transfer efficiency is increased due to both the heat carried by bubble motion and the thermal convection of the liquid film. (D) Demonstrated liquid film boiling heat transfer on a hierarchical mesh-covered (hi-mesh) wicking structure. Nucleated bubbles can rapidly grow and escape from the hi-mesh wicking structure by bursting on the capillary liquid film, frequently fluctuating the liquid film to increase convection. Reprinted with permission from (Wen et al., 2018b). Copyright (2018) Elsevier. (E) Significantly enhanced heat transfer performance was demonstrated on the hi-mesh wicking structure: enhanced CHF and HTC compared with capillary evaporation on mesh wicking structure (Wen et al., 2018b) and enhanced HTC compared with nucleate boiling on nanowired surfaces (Wen et al., 2017a). The heat transfer enhancement of liquid film boiling strongly indicates the success of the liquid film boiling strategy for enhancing evaporation. Published by Elsevier. (F) Comparison of HTC and CHF of various evaporation/boiling heat transfer processes.

Improving the CHF and HTC in evaporation/boiling processes with mini-, micro- and nanostructured surfaces has been a long pursuit for many decades (Allred et al., 2018; Chen et al., 2009; Li et al., 2008; McGrew et al., 1966; Wang and Chen, 2018; Wen et al., 2017a, 2018a). Heat transfer performance of thin film evaporation on the wicking structure is highly dependent on the liquid film thickness and liquid-vapor evaporation area (Figure 3A). However, the CHF of capillary evaporation is greatly limited if there is no enough liquid to rewet the heated area (Hanks et al., 2018; Plawsky et al., 2014). Intensive efforts have been devoted to developing various hierarchical micro/nano-structured surfaces and functional coatings to enhance evaporation heat transfer by either reducing liquid film thickness (for increasing HTC) or increasing evaporation area (for enhancing heat flux) (Adera et al., 2016; Plawsky et al., 2014). However, it is still challenging to significantly enhance both CHF and HTC on the same structured surface due to the nature of the capillary evaporation process which has conflicting requirements on liquid film thickness. For example, a thicker liquid film tends to delay surface dryout with higher CHF while a thinner liquid film is preferred for low thermal resistance for evaporation (higher HTC). On the other hand, as shown in Figure 3B, for nucleate boiling which occurs on a heated surface submerged in a liquid pool, the vibrant growth and departure of bubbles can directly carry heat away from the solid surface (Li et al., 2008). As nucleate boiling becomes more intense at high heat flux, neighboring bubbles can merge and chaotic two-phase interactions occur. Although the transient nature of bubble dynamics on the heated surface makes it difficult to accurately account for the contributions from each mechanism, *e.g.*, micro-convection, macro-convection, and microlayer evaporation, the heat transfer performance of nucleate boiling is essentially determined by bubble activity and bubble-induced convective liquid flow, *e.g.*, the nucleation, growth, coalescence, and departure of the bubbles (Dhillon et al., 2015; Li et al., 2008; Wen et al., 2018a). Manipulating the dynamical behaviors of bubbles on the surfaces, *e.g.*, increasing bubble nucleation sites, extending tri-phase contact line of growing bubbles, accelerating bubble detachment, and bubble-induced convective liquid flow can enhance the HTC and CHF of nucleate boiling in a liquid pool. Strategies have been employed to increase solid-liquid contact area by increasing surface roughness (Chu et al., 2012), to improve bubble formation by increasing microcavities (Li et al., 2008) or decreasing nucleation energy barrier (Allred et al., 2018), and to separate liquid-vapor pathways for uninterrupted liquid supply and bubble removal (Jaikumar and Kandlikar, 2016). However, it is very difficult to change the buoyancy-controlled bubble departure from a heated surface for nucleate boiling in a liquid pool, commonly with a departure diameter of several millimeters for water, leading to a limit on the CHF and HTC (Cho et al., 2016; Kandlikar, 2019; Wang and Chen, 2018; Wen et al., 2018a).

Maintaining sufficient liquid supply to wet the heated area and maximizing heat dissipation across the liquid fluid are the key to enhancing both CHF and HTC of evaporation and boiling processes (Plawsky et al., 2014; Sudhakar et al., 2020; Wen et al., 2018a). It is thus desirable to have a surface that has high capillarity and high permeability to spread the liquid over the heated area for large CHF while minimizing the overall thermal resistance across liquid film for high HTC. To reduce conduction thermal resistance of a liquid film that determines the HTC in capillary evaporation, dynamic bubbles are activated in a thick capillary liquid film which also allows low resistance liquid supply along with the wicking structure for high CHF (Figure 3C) (Ćoso et al., 2012; Weibel et al., 2010). The dynamic behaviors of the bubbles, *e.g.*, nucleation, growth, escape, and bursting, can frequently disturb liquid film that is confined within the wicking structure, increasing convection heat transfer through the liquid film (Ćoso et al., 2012; Palko et al., 2015; Sudhakar et al., 2020; Weibel et al., 2010; Zhang et al., 2018). The bubbles formed in the wicking structure can also increase additional liquid-vapor interfaces for liquid vaporization near the tri-phase contact line of the bubbles and carry heat away directly from the heated substrate by bubble growth, motion, and bursting. To facilitate the coupling of bubbles and a liquid film in a porous structure, defined as liquid film boiling, a wicking structure should have high rate flow passages for liquid supply to the heated area, plenty of effective nucleation sites for bubble formation, and sufficient bubble channels for vapor escape.

A recent work on capillary-driven liquid film boiling on a hierarchical mesh-covered mesh (hi-mesh) wicking structure has demonstrated the effectiveness in coupling bubbles with a liquid film for liquid film boiling (Figure 3D) (Wen et al., 2018b). The hi-mesh wicking structure is formed by stacking multiple layers of woven copper mesh on a flat substrate, resulting in plenty of interconnected microchannels between woven mesh layers for transporting fresh liquid to wet the heated area. High-density microcavities with a typical size of 3-10 μm are etched on the mesh wire surfaces, which increase effective nucleation sites to promote bubble formation in the wicking structure at a small superheat (Li et al., 2008). When the heat transfers from the substrate to hi-mesh, nucleated bubbles can rapidly form in the microcavities at a small superheat of 4.2 K and escape from the hi-mesh wicking structure through the pores among the mesh wires. With a typical diameter in several hundred micrometers, the growing bubbles burst on the surface of capillary liquid film, which frequently fluctuates liquid film in the hi-mesh structure to increase convection heat transfer (Sudhakar et al., 2019b; Wang and Chen, 2018; Weibel and Garimella, 2012; Wen et al., 2018b). A significant heat transfer enhancement in both CHF and HTC has been demonstrated on the hi-mesh wicking structure with a large area of 100 mm^2^ due to the dynamic bubbles introduced to capillary liquid film (Figure 3E). Compared with conventional thin film evaporation and pool boiling (Figure 3F) (Plawsky et al., 2014; Wen et al., 2017a, 2018b), liquid film boiling provides a more superior liquid-to-vapor phase-change scheme with a higher CHF and HTC due to the increased liquid supply capability and reduced effective thermal resistance of a liquid film by activating the ONB at a smaller superheat.

## Summary and outlook

After nearly a century of intensive efforts in studying the physical mechanisms and exploring various strategies to enhance phase-change heat transfer processes, questions are now being centered around what are the limits in minimizing droplet removal size for dropwise condensation, maximizing liquid vaporization area for capillary evaporation, and accelerating bubble departure for pool boiling. While challenging to address these questions directly, there are exciting promises when using micro/nano-structured surfaces and functional coatings. By coupling droplets/bubbles with a liquid film, the effective thermal resistance between the liquid-vapor phase-change front and the solid substrate is significantly reduced during phase-change heat transfer. For condensation, using a vapor-permeable layer bonded onto a hydrophilic porous structure, not only allows for rapid vapor condensation on a controlled thin liquid film but also enables continuous condensate removal from the surface in the form of falling droplets. Benefiting from the low conduction thermal resistance of thin liquid condensate-solid composite layer, sustainable heat transfer enhancement has been demonstrated on the structured surfaces, *e.g.*, hi-mesh surface and biphilic surface with different wettability (Oh et al., 2018; Wen et al., 2018c). For evaporation/boiling, dynamic bubbles are activated in the wicking structure to promote convection of capillary liquid film, to enlarge the evaporation area near the tri-phase contact lines of the bubbles, and to accelerating vapor escape by bubble bursting at the liquid film surface. Such a hybrid evaporation-nucleate boiling mode in a capillary liquid film, referred to as liquid film boiling heat transfer has demonstrated both high CHF and HTC (Ćoso et al., 2012; Sudhakar et al., 2019a; Sudhakar et al., 2020; Weibel et al., 2010; Wen et al., 2018b).

Enhanced heat transfer performance has been demonstrated by the novel strategy that couples droplets/bubbles with a liquid film. However, many opportunities and challenges remain for further exploration and deployment, including fundamental understanding of the two-phase flow dynamics, enhancement in increasing fluid transport and decreasing thermal resistance, and improvement in surface durability and system design for real-world applications.

• Current understanding of droplets/bubbles coupled with a liquid film is essentially based on individual droplet/bubble behaviors, which are applicable under low heat fluxes. Further research is needed to understand complex two-phase flow dynamics and associated heat transfer mechanisms under high heat fluxes leading up to CHF, *e.g.*, bubble jetting phenomena in the wicking structures, liquid condensate overflow on the top of vapor-permeable layer, and vigorous crossflow of bubbles/droplets and a liquid film. Partition theory has been used to analyze droplet/bubble behaviors in phase-change processes for a long time, which helps identify the individual mechanism. It would be useful if the interplay between microlayer evaporation/condensation at the tri-phase contact line, droplet/bubble motion, and liquid film convection is clearly understood so that effective strategies to enhance liquid film heat transfer could be developed. Another area for research is to clarify the thermodynamics of bubble nucleation in a thin capillary liquid film under a small surface superheat, which is critical for surface design on effective nucleation sites and wicking structure thickness.
• Combining hybrid wettability and surface features in different length scales can promote both the formation and departure of droplets/bubbles in a wide range of surface subcooling/superheat, as well as preventing flooding phenomena of droplets/bubbles. Further improvements in liquid film flow, *e.g.*, accelerating liquid condensate removal or increasing fresh liquid rewetting, can be implemented by gradient and directional surface structures, such as activating self-driving liquid transport by anisotropic structures and guiding capillary liquid flow by nonuniform structures. Counterflow of vapor diffusion and condensate removal through non-wetting permeable layer, *e.g.*, hydrophobic membranes and meshes, also need to be optimized to prevent liquid condensate flooding and to maximize vapor flow across the porous layer. Addressing the crossing two-phase flow in the direction perpendicular to the heated wicking structure that is liquid wicking flow and bubble escape is important for future exploration in liquid film boiling heat transfer, especially under high heat flux with the formation of excessive bubbles. More attention should be paid to low surface tension fluids, such as hydrocarbons or refrigerants, where filmwise condensation is commonly formed. As an effective approach, the lubricant-infused surface has been explored to promote dropwise condensation of low surface tension fluids for heat transfer enhancement. The coupling scheme proposed here can also significantly reduce the conduction thermal resistance of condensate film. A non-wetting porous layer with higher liquid repellency is necessary to create a Laplace pressure barrier for confining condensate film of low surface tension fluids on the substrate.
• Benefiting from the advance in phase-change enhancement using micro/nano-structured surfaces and functional coatings, heat transfer efficiency and capability of many thermal devices have been significantly improved in the lab-scale in the past decade. Stepping away from fundamental heat transfer enhancement and lab-scale devices, several aspects on the surface durability and system design need to be addressed for pushing the frontier of enhanced surfaces into large-scale industrial implementation. Simultaneously achieving stable liquid wicking flow on a high energy capillary surface with good anti-pollution to the adsorption of organic and oil molecules has been one of the most challenging tasks in the field. The fouling and corrosion of structured surfaces can also change initial structure morphology due to the crystalline deposits of inverse-solubility salts or chemical reactions, resulting in the degradation of heat transfer performance over time, especially for the wicking structures with high surface energy (Attinger et al., 2014; Hanks et al., 2018). In addition to surface durability, applied studies on the optimization of fluid flowing and equipment structure in the system-level are essential to enable the integration of heat transfer surfaces into real devices and systems.
